# The American Dental Association

**Published:** 1882-08

**Authors:** 


ARTICLE II.

### THE AMERICAN DENTAL ASSOCIATION.

### ITS LATE MEETING AT CINCINNATI.

The twenty-second annual meeting of the American Dental Association was held in Cincinnati, commencing on Tuesday morning, August 1st. There was a large number of members in attendance, the sessions were harmonious, and although no papers of unusual import were presented, yet the meeting may be said to have been more than usually successful. The profession of Cincinnati extended their hospitality in a very liberal manner, and there was a considerable accession of new members to the Association, some of whom will undoubtedly become permanent and useful associates. Cincinnati is not the very best place in America for estivation. August days are apt to be warm ones ; the grasshopper becomes a burden, and the desires of man fail—if we except the constant longing for cooling drinks and clean water for a refreshing bath. It was not built for refrigerating purposes, but lies, like Truth, at the bottom of a well, while from the summits of the surrounding high hills one can peer down upon the black clouds of smoke and soot which hide the great city below—can even catch evanescent glimpses of the spires and domes which tower above the great manufactories and the busy marts of commerce, lost to his gaze in the haze and mist and dark obscurity of their own intense activity. In truth, Cincinnati is not without suspicions of dirt; and linen does not long retain the glossy pristine whiteness with which it leaves its native Trojan laundry. Calico shirts and dark lawn ties are, for good and sufficient reasons, exceedingly popular there, while Marseilles vests and duck suits are at a discount. In short, Cincinnati is a great, busy, dirty, practical, enterprising, business city, that is not without its as yet rather unsatisfied and unsatisfactory æsthetical longings. The meeting, happily for the members, was held upon the summit of one of the surrounding hills, though all were compelled, because of the lack of sleeping accommodations, to return to the city at night, where darkness was under our feet, and the pavilion round about was dark waters and thick clouds of the skies. The officers for the year were: H. A. Smith, of Cincinnati, President; W. C. Barrett, of Buffalo, First Vice-President; G. J. Friedrichs, of New Orleans, Second Vice-President; G. W. Cushing, of Chicago, Secretary; A. M. Dudley, of Salem, Correspondent; and W. H. Goddard, of Louisville, Treasurer.

### FIRST SESSION.

The meeting opened at the Highland House at 11 o'clock A.M. of Tuesday, August 1st, with prayer by Dr. W. H. Morgan, of Nashville. Dr. A. Berry, on the part of the profession and citizens of Cincinnati, then delivered an address of welcome, in which he alluded to the greatness and glories of Cincinnati as a city, to the rapid and steady growth of dentistry in usefulness and in the estimation of the people since the last meeting in Cincinnati, sixteen years ago, to the fact that the second dental college in the world was here established, that here had its origin the Mississippi Valley Dental Association, for twenty years past the oldest dental society in the world, and he closed by bidding the society and its members a hearty welcome to the city.

Dr. W. C. Barrett, of Buffalo, was called upon by the President, and requested to make response. He said that while all were more or less familiar with the glories of Cincinnati, while all had at least heard of the enterprise and greatness of the city, had been told of its beauty and loveliness, of its public buildings and halls of learning, yet those present had gathered, some of them from the remotest corners of our country, for something else than mere admiration. They had come for the purpose of looking over the field of dental science, of exchanging views and mutually helping each other. Nor did they come as strangers. Science knew no boundary-lines—no city's ramparts, no country's walls, nor rivers, lakes or oceans, sufficed to stay the onward march of scientific investigation. Wherever students met there was their scientific home, and all were brothers. The members were proud to accept the welcome of Cincinnati dentists, because among them were found men whom the profession everywhere delighted to honor. The Association came to Cincinnati with high hopes of a profitable and pleasant session, and the speaker doubted not that their expectations would be more than realized.

Drs. G. L. Field, T. L. Buckingham, and J. Taft were appointed a committee to draft suitable resolutions upon the death of Dr. D. C. Hawxhurst, a former member of this Association, who died in Paris, last winter, a martyr to his love for scientific study.

On motion of Dr. Barrett, the Secretary was instructed to telegraph the sympathies and best wishes for a speedy recovery, to Dr. M. H. Webb, of Lancaster, who was then lying seriously ill at his home.

Drs. J. N. Crouse, L. D. Shepard, and C. N. Pierce were appointed a committee to draft suitable resolutions upon the death of Dr. M. S. Dean, of Chicago, a former Secretary and President of the Association.

The Constitution was amended by the adoption of an article offered by Dr. Shepard, last year, and which reads as follows : Section 2. The officers may, for extraordinary reasons, change the time and place of meeting upon the written consent of ten of the fifteen members of the Executive Board.

### SECOND SESSION.

Over one hundred members had arrived when the afternoon session was called to order, and there was also present a large number of visitors.

Dr. Barrett was called to the chair, and Dr. Smith, the President, delivered the annual address. He spoke of the flourishing condition of the Association, and of the work in which it was engaged. He paid a glowing tribute to the memory of Dr. M. S. Dean, and then proceeded to consider the work of the sections. He referred especially to Section Seven, Physiology and Etiology, and the great importance of an exhaustive study of the causes of dental diseases. He thought there was a tendency upon the part of some of our best men to choose for their fields of labor sections which did not present the difficulties here found, and he urged more faithful work, especially in Etiology. To encourage this he recommended that the sum of two hundred dollars be set apart by this

Association as a prize to be awarded the best paper, based upon strictly original investigation, and devoted to a study of the causes of decay in teeth.

Reports from the different sections were then called for, but none were ready until Section Three, Dental Literature and Nomenclature, the last upon the list for this year, was reached, when Dr. W. H. Atkinson, the Chairman, took the floor and said that but one paper had been presented, and that was a continuation of the subject of Nomenclature and Terminology as presented in previous years by himself. The paper was read by the author, and it raised a decided breeze. He had endeavored, among other things, to show the homology which existed between the tones of the voice and the tissues of the body.

Dr. Friedrichs demanded of the essayist how it was possible for the tones of the voice to correspond with the tissues of the body. He said there could be no such thing as a universal language, unless music was its expression.

Dr. Atkinson, implying that the speaker was but the mouthpiece of others, said that second-hand cheese was sometimes maggoty and sometimes mouldy, when originally it had been pure and nutritious.

Dr. George Watt said that the paper was a mass of loud-sounding nothingness. The whole matter was a vagary, a chimera, and he moved that it be referred to a special committee of three for proper consideration and report as to its fitness for presentation here.

Dr. Rehwinkle said that life was too short to waste on such a subject. Why should dentists try to build up a new language? Year after year the essayist had been striving to hammer this matter into our heads, and he had not been able so far to make one man understand him. They might all be blockheads in the estimation of the essayist, but while they could fathom other abstruse subjects, this one did not present the first handle of good sense for any one to catch hold of.

Dr. Atkinson warmly responded and defended the paper and subject, at times decidedly carrying the war into the enemies' country.

Dr. Barrett regretted that such an unpleasant discussion should have occurred. He said that the majority of the members attended the meeting for another purpose than to indulge themselves, or listen while others indulged in personal thrusts, and he moved that the whole subject be laid upon the table.

Dr. Watt and Dr. Atkinson then proceeded to explain their remarks, in the course of which each dealt out considerable of what non-scientific people call "taffy;" but the former insisted that while the subject of the paper had been philological, the matter had not been nomenclature. For the last four years the society has patiently partaken of this hash without having been able to extract from it any nutriment whatever. And now came a continuation of the subject, with the same *animus* from the same *animile.* Where was this thing to end? There was not an original thought in the paper. It was but the second-hand vaporings of Stephen Pearl Andrews. Hence he would insist upon his motion, that it might be decided whether the society records should still be burdened with this useless matter. He did not care what the committee decided to do, whether to send it to the benighted Patagonians or to have it bound in calf, so long as they definitely disposed of it. Perhaps the best thing would be to send it back to its first cause, the original author. But in anything which he said he declared that he was only striving to do honor to Dr. Atkinson. He would have it referred to a special committee as a very special way of honoring the paper. He was one of the original discoverers of Dr. Atkinson's greatness, to which he wished to pay special homage.

After considerable desultory discussion and sharp firing, the air cleared somewhat, and it was voted to refer the paper to a committee of five, which was finally, after a number of members had declined the appointment, made up of Drs. G. W. Keeley, G. J. Friedrichs, M. H. Odell, F. H. Rehwinkle, and C. N. Pierce.

Dr. J. N. Crouse said that Dr. Atkinson had not been fairly treated. It was beneath the dignity of this Association to make fun of any report that might be presented. He would give the meanest dog in town a chance for his life. It was a new and unheard-of thing to send the report of a section to a special committee in this way. It was intended as a mark of degradation, and he was opposed to it.

Dr. Watt replied that the speaker was setting up a man of straw, and then trying to demolish it. The committee was still under the control of the Association.

Dr. Atkinson, in conclusion, reminded the Association that he had been the only chairman of a section who had come prepared with a report. He had been entirely honest and conscientious, and as far as ability went he felt himself able to cope with the best of them. He was a believer in immediate inspiration, and he usually tried to walk as he was inspired to walk, and to present whatever it was given him to say. All he asked was a fair chance, and the decent consideration due to the magnitude of his subject. That it was abstruse and beyond the comprehension of unilluminated blockheads he freely conceded, but he was fully persuaded it was the great question of the hour. He would cheerfully acquiesce, however, in any decision to which the Association might arrive.

THIRD SESSION.

Time was given on Wednesday morning for meetings of the section, and the Association was not called to order until ten o'clock. A considerable number of members had arrived since the last session, and fully one hundred and fifty delegates were now present.

Dr. W. H. Goddard presented a resolution providing that, after that date, the Treasurer of the society should be required to give such bonds for the security of the funds in his possession as the Executive Committee of the second section should require, and that his predecessor should not surrender any of the property of the society until such bonds be executed.

Dr. Goddard, the Treasurer, said that during the sixteen years in which he had held the office there had been no security required of him, and as he proposed to relinquish the position he thought some check should be placed upon his successor. The resolution was adopted.

The privileges of the floor were extended to Prof. Chas. Mayr, of Springfield, Mass., and to Prof. Stubblefield, of Nashville, Tenn., who responded in pertinent remarks.

The committee to whom had been referred the paper of Dr. Atkinson made the following report:

"The committee to whom was referred the paper on Philology, read by Dr. W. H. Atkinson, as Chairman of the Section on Dental Literature and Nomenclature, would respectfully report that they have carefully considered the subject referred to them, and are of the opinion that the paper under consideration is foreign to the purpose of the section under which it was read, and that if the Association desires further communication upon general philology a section of that title should be created ; but in view of the fact that at previous meetings of this Association similar papers have been received without adverse criticism, they would suggest that this one be allowed to take the usual course, and go with other reports to the Publication Committee."

The report was adopted.

Section Four was called upon, and the Chairman, Dr. E. T. Darby, read a brief report, and presented a paper upon the Regulation of Teeth, which was read by its author, Dr. W. N. Morrison.

In the course of the paper the author said that in the whole course of his somewhat extended experience in regulating teeth, he was not aware of having made but one mistake, and that was in the case of a small, poorly developed, diminutive patient, with large, well-developed teeth. He was free to admit that in this instance it was probably a mistake to attempt to save all the teeth, as the result proved that the mouth was too prominent. But this was only one case. There are thousands of instances at the other extreme. The public was clamorous for deformed mouths, and the dentists were fostering this vitiated taste by an almost indiscriminate extraction for the purpose of regulation. The consequence was that we were fast assuming a narrow, pinched, hatchet-shaped, or flat, cadaverous expression, until the thin-faced Americans were becoming a byword among nations. It was a disgrace to the profession that there were so many who claimed to be intelligent, progressive dentists, who yielded to the demands of patients, and practised such opprobrious dentistry. A few months ago the public went into ecstasies over Patti's small mouth and beautiful teeth, when in realitv it was an actual deformity—teeth irregular, and one canine so much out of the arch that her lip sometimes got hung upon it. Her profile had that sorrowful, pinched appearance which is so common among our people. Several years ago the newspapers had given an account of the selection of a typical American female head, by the designer of the new silver dollar, that should accurately represent the correct type of American beauty. He did not know the lady nor her dentist, nor had he any information about her teeth or their articulation, but seeing her face upon the silver dollar, he appealed to any intelligent dentist to know if he took any risk in venturing the assertion that her mouth did not contain thirty-two teeth. The upper lip appeared as if she had been in the hands of one of those dentists who extracted teeth for regulating purposes.

Teeth are easily moved if the force be properly applied, but they also readily return to their original positions. He therefore had decided objections to all the complicated apparatus of levers, screws, inclined planes and wedges, so freely urged by some dentists.

He depended mainly upon rubber ligatures, and to secure the proper direction and guidance he made thin platinum bands to fit the tooth. A hook or an eyelet was soldered to this, and then the band was fastened to the tooth by cementing it with oxychloride of zinc, and this remained until the operation was entirely completed.

The essayist described his method of procedure in typical cases, and was listened to with the closest attention throughout.

Dr. Atkinson thought there was no material really adapted to cases of regulating, except Reese's metallic plates. He detailed several illustrative cases, and exhibited the plates which were used.

Dr. McKellops said that when he was in London last summer, he devoted considerable time to a study of Mr. Coffin's method of regulating by means of piano wire. He saw twenty-five hundred plates which had been in actual use, and the most astonishing results had been obtained in the very briefest time. His own son, a lad of twelve years, was with him, and he had a cuspid which all the apparatus he had been enabled to apply had not been able to bring into place. Just before he left London Mr. Coffin made and inserted a plate for the boy, and although it had received no attention whatever, when they arrived in New York the tooth was occupying its proper position. When the boy was sea-sick he would slip the plate out, render up his accounts, and then put it back again. It was instantly and easily removed, and as easily reinserted. Dr. McKellops exhibited some of the plates and explained their great advantages.

Various devices, apparatus, and methods for regulating teeth were presented, and their merits discussed by Drs. Field, Pierce, Shepard, Darby, Noel, Eames, Butler, Dorrance, Keeley, Crouse, and others, and the discussion occupied the remainder of the time until the hour for adjournment.

FOURTH SESSION.

Dr. G. L. Field, Chairman of the committee appointed to present suitable resolutions expressive of the sense of the Association upon the death of Dr. D. C. Hawxhurst, presented a report which was ordered spread upon the minutes.

Section Five was called, but in the absence of the Chairman no report was presented.

The report from Section Six, Pathology, Therapeutics, and Materia Medica, was presented by the Chairman, Dr. F. M. Odell. Two papers were recommended to the consideration of the society; one on Pathology, by Dr. A. O. Rawls, of Lexington, Ky., the other upon Therapeutics, by Dr. A. W. Harlan, of Chicago. Both papers were read, and discussed by the members.

Prof. Mayr asked Dr. Rawls what he meant by the term *vis vitæ*, or vital force. The reply was, that it was applied to some mysterious power, but what that power was it was hard to tell. Prof. Mayr said it was an empty word, and meant nothing whatever.

The discussion here took rather a desultory turn, and led toward a consideration of the doctrine of Materialism, in which Drs. Buckingham, Morgan, and Watt took occasion to inveigh rather strongly against the skeptical tendencies of modern scientific research. Prof. Mayr, Drs. Barrett, Rawls, and others, declared that in the study of physics they proposed to follow wherever truth led them, regardless of any creeds or doctrines, formulated perhaps long before the knowledge of scientific study or the teachings of unerring scientific truth had revealed the history of the ages.

Dr. L. C. Ingersoll, of Keokuk, explained the difference between salivary and sanguinary calculus.

Dr. George Watt replied that he could not make out what the gentleman was driving at. He thought that he must have made some of his assertions just to try his wind, and find out if he was in good discussive condition. What he called sanguinary calculus had long been known to the medical profession, but they had never recognized the necessity for a new name for it. All calculi came from the blood. He had once taken one weighing thirteen ounces from the abdominal region ; but he didn't call it a *sanguinary* calculus.

Dr. Buckingham recommended as a bleaching agent for the teeth a recipe of Dr. Kirk—sulphide of soda with borax.

Dr. Atkinson took the floor and said that he was sorry to see that this body was getting as crazy and as "Atkinsonian" as he was himself. They were all at sea because of ignorance of the laws of terminology. What was ulceration but suppuration—suppuration but inflammation—inflammation but oxydation ? He was a firm believer in the *vis vitæ* and in inspiration, and both he believed were the gift of God.

At half-past four the Association adjourned to accept an invitation to attend an organ concert by Prof. Whiting, at Music Hall, in the College of Music. The members took street cars at the door, and were conveyed down "the incline" and across the city to the college, where Prof. Geo. E. Whiting exhibited all the resources and powers of the great organ in a programme of classical music, to which it was an unexpected pleasure to listen. While he was playing "The Storm Fantasia," by Lemmens, a real storm of wind and violent rain set in, which lent a naturalness and realistic sense to the performance that made a decided impression upon the listeners.

FIFTH SESSION.

The consideration of Section Six was resumed, and a paper upon Diathesis and Cachexia was read by Dr. F. M. Odell. It proved to be of great interest, but was one of those essays which it is exceedingly difficult to epitomize, requiring a presentation of the whole to give one a clear idea of its completeness. It was discussed by Drs. Atkinson, Friedrichs, Buckingham, Watt, Kulp, Darby, and Prof. Mayr.

Dr. J. N. Crouse, from the committee appointed to draft resolutions expressive of the feeling of the Association upon the death of Dr. M. S. Dean, read a brief sketch and eulogy of the life of Dr. Dean, and a series of resolutions, which were adopted and ordered to be engrossed upon a memorial page in the Secretary's minutes of the meeting.

Section Seven—Physiology and Etiology—was called, and Dr. W. C. Barrett, the Chairman, read a brief report, in which he stated that the section appeared at a disadvantage, because it was only last year set apart from Section Five, which had been thought too comprehensive, and as but few members elected to work in it, there had been no formal organization. The physiological condition of dental tissues and organs must first be understood before it was possible to comprehend any pathological changes. The etiology, or origin of oral diseases, was a subject to which far too little attention had been directed. In weight and importance of the subjects to be considered, this section ranked all the rest, but to accomplish anything required hard study and close observation through the whole year. As a consequence, where one dentist elected to be enrolled upon its register of active workers, ten chose the easier fields of education, or operative dentistry, which required from their members no particular study or preparation; fields in which a creditable record might easily be made, and in which there was opportunity for unlimited talk and discussion without much hard work.

That part of the President's address which recommended a prize for the best essay on the Etiology of Dental Decay, had been referred to this section, and the Chairman offered a resolution that the sum of two hundred dollars be set apart to be awarded by a special committee of three members, to be appointed by the President, to the author of the best paper upon the origin of tooth caries, the paper to be based upon original investigation, and when read to become the property of the Association. Competition should not be confined to members of this Association, but should be open to the world. The resolutions prescribed the conditions upon which the prize should be awarded, and in certain contingencies divided.

The only paper presented from the section was one upon "The Origin and Physiology of Nervous Force," by Dr. W. C. Barrett, which was read. The essayist took up the doctrine of the unity of the so-called physical forces, and their correlations. He showed that as chemical force was, in the galvanic battery, changed into electricity, and electricity into light in the incandescent arc, and in the galvano-cautery into heat, these forces were all one, though differently manifested. He then argued that while all these different manifestations were thus correlated and in perfect harmony, it was absurd to suppose that in the higher involutions of nature all should be discord and clashing. Nervous force he held to be but another mode of manifestation of the parent force; that as the molecular movements and changes which we call chemical affinities, in the battery cell result in the evolution of electricity, so the similar molecular changes which we call nutrition, or digestion and assimilation, in our bodies result in nervous force. In the gymnotus or electrical eel we see nervous force transmuted into electricity. In the common firefly it is changed into light. He detailed the results of numerous experiments and vivisections, in which he had seen electricity, heat, and light transfused into nervous force.

The paper was discussed by Drs. Atkinson, Buckingham, Watt, and others.

Considerable routine business was transacted, and an opportunity was offered for members to indicate to what section they desired to be assigned.

SIXTH SESSION.

The order of business for this session was the election of officers, and the selection of a place for meeting next year. Niagara Falls, Nashville, Cleveland, Pittsburgh, and Atlanta were named. On the third ballot Niagara Falls received a majority of the votes cast, and was declared chosen. The society then proceeded to an election for officers for the ensuing year, which resulted as follows:

President, W. H. Goddard, Nashville, Tenn.; First Vice-Pres., Geo. J. Friedrichs, New Orleans, La.; Second Vice-Pres., Edwin T. Darby, Philadelphia, Penn.; Recording Secretary, Geo. H. Cushing, Chicago, Ill.; Corresponding Secretary, A. W. Harlan, Chicago, Ill.; Treasurer, Geo. W. Keely, Oxford, Ohio; members of Executive Committee, Geo.

L. Field, Detroit, Mich. ; A. M. Dudley, Salem, Mass. ; J. N. Crouse, Chicago, Ill.

The society then accepted an invitation from the dentists of Cincinnati to visit the Zoological Gardens, where they would be suitably entertained. Street cars were at the door of the Highland House, which conveyed the members of the Association and invited guests to the depot of the Cincinnati Northern Railroad, where all embarked for their destination. After their arrival some time was spent in examining the animals and visiting the gardens, and about six o'clock the members gathered at the restaurant, where had been provided a substantial and elegant lunch. Over two hundred guests sat down to the tables and listened to the music of the orchestra, which was in the gallery. After the tables had been pretty thoroughly cleared, the Chairman of the Committee of Arrangements proposed a number of toasts, and called upon members to make response. Dr. Goddard, the President elect, who was first called upon, congratulated the Association upon having enjoyed one of the most pleasant meetings which it had ever held. He believed he was now its oldest member, and he could not recall a more successful meeting during the whole of its existence.

Dr. W. C. Barrett was next called upon, and said that dentists were hard-working men ; whatsoever their hands found to do they did with all their might, and the present condition of these tables bore evidence of the truth of his assertion. There was no class of men who needed to use greater caution in the conservation of their nervous force, and he was glad to be able to say that there was no set of men who really enjoyed such a social time as they had been privileged with that evening, better than dentists. Every man was going home with his stomach and his heart full of the hospitality of their liberal Cincinnati brethren.

Dr. J. Taft eulogized the President elect, who had for so many years been unfailing in his attendance upon the meetings of this Association. Now when he was nearly ready to lay down all professional burdens, it was but meet and fit that the culmination and close of his professional life should be in his election as President of the Association, which for sixteen years he had served as Treasurer.

Dr. L. C. Ingersoll said that the success of dentistry to-day was the result of one hundred years of steady professional progress, one advance growing out of another.

Prof. Charles Mayr declared that he did not know which he had enjoyed most ; the social or the professional features of the meeting. A

foreigner as to nationality, he was glad of the opportunity to attend this meeting of the Association.

Dr. Atkinson said he was happy in contemplating so many beneficent results from this meeting. He felt sure that some of the gray heads then present might with justice claim some credit for the progress made by the dental profession. Dentistry had not been recognized by the medical profession, and he, for one, rejoiced thereat, for it had resulted in our advance. He did not wish recognition at the hands of the old women who made up the great body of medicine.

Drs. W. W. Allport, W. H. Morgan, T. L. Buckingham, and G. W. Keely, the Treasurer elect, were called upon, and briefly responded. But long before all the speeches were ended some of the younger people were enticed by the sweet music of the orchestra to open a dance, which proved so alluring that it was not long before some of the grayest, gravest, and most reverend heads were bobbing up and down in a quadrille, and feet that had for many a lustre been accustomed to but sober paces were, under the inspiration of the occasion, flying through the mazes of the waltz.

Later in the evening the grounds were brilliantly illuminated, and the orchestra gave an out-of-door concert. It was late before some of those who were accustomed to early hours sought their pillows that night, and many a surmise was indulged in that hats would have all too limited accommodations for their owners' heads the next morning.

SEVENTH SESSION.

Section One was called; Artificial Dentistry, Metallurgy, and Chemistry. In the absence of the Chairman, no formal report was presented. Dr. W. H. Dorrance read a paper on the evil results following the use of vegetable or organic bases for artificial teeth. The paper set forth the harm caused by rubber and celluloid, and claimed that nothing but metal should be used in the mouth. So conclusive were the arguments adduced that a motion was made at the close of the reading that a sufficient sum for the printing and free distribution of the paper be set apart by the Association. At Dr. Dorrance's request the matter was deferred one year, that he might have time to rewrite and condense the paper.

Dr. Dorrance presented some of his alloy for making gold or silver solder. He takes of silver one part, zinc two parts, and copper three parts. The silver and copper are melted together, and the zinc added in small pieces until the fumes disappear. With this alloy the gold is mixed to the proper degree of fineness, and the whole poured into an ingot and rolled into a plate.

He also presented a compound gas blow-pipe, which he had used for eighteen or twenty years. He claimed nothing specially original in it, yet the first one he made contained the same principles now used in Fletcher's and many other blow-pipes which have since been patented.

Different dentists inquired if these blow-pipes could be bought. Dr. Dorrance answered that none had ever been made, save for his own use, and occasionally one which he had presented to a friend. The one exhibited he had made for Dr. Barrett, and he had no others.

On motion it was declared the sense of the society that the blow-pipe was meritorious, and that Dr. Dorrance be requested to manufacture it for sale, if he could, consistently with other duties, do so.

The resolution offered in the report of Section Seven, setting apart the sum of two hundred dollars for a prize essay fund, was taken up and adopted. The President announced that he would name the committee at a later date.

It was moved that the sum of three hundred dollars be set apart to assist in examining and tabulating certain collections of skulls in the museums of the country, with a view of collecting data for the proper study of dental cases.

An animated debate ensued, the importance of the undertaking being urged upon the one hand, while in opposition it was declared that it was mere foolery and travesty upon science to commence such a work until it could be performed by men of scientific training; that the proposed movement would not only bring results of no value, but any report would be positively misleading, unless some recognized student was at the head of it; that such was not the case in this instance, and hence the proposal was a mischievous one, notwithstanding the importance of the subject.

The vote was at first a tie, but the motion was finally lost by one member announcing a change of his vote from aye to no.

### EIGHTH SESSION.

Section Two, Dental Education and Literature, was called. Dr. J. N. Crouse made a brief report upon the confusion in the status of different dental educational institutions, and urged action in the direction of fixing a standard by which colleges might be judged. There was, he said, an increase in the number of colleges out of all proportion to the increase in students.

Dr. Atkinson pleaded for more pluck and honesty on the part of colleges. The idea of granting a diploma solely upon the ground of time spent in the schools, he characterized as born of darkness and iniquity.

Dr. Pierce said there could never be a high standard of education until there is a board of examiners, independent of the teachers, who alone shall have power to grant diplomas. He criticised Dr. Atkinson's idea of granting a diploma to any man upon his showing his fitness, without regard to time spent in the schools.

Dr. Barrett said that the idea of granting a diploma upon the showing of merit, is in theory very alluring and beautiful. But Wisconsin has shown what a dental college professedly founded upon those principles can be guilty of. Every diploma mill is ostensibly opened for the purpose of graduating students when they are qualified, and not at the end of a fixed, arbitrary term. They very plausibly urge that this is the only fair way to treat students—to graduate them on their merits—but it must be understood that the faculty propose to be the judges as to the merits of the students, and their criterion is the ability to pay the fees demanded. The proposal to confer a diploma when the pupil was entitled to it, without reference to time, looked very fair, but it had been the plea under which every fraudulent diploma, the sale of which had been a national disgrace and a professional shame, had been issued.

Dr. Taft hoped the agitation of this subject would be continued until the time should come when state examining boards would be appointed, who alone should be authorized to graduate pupils. Until that time came the scandal of unearned diplomas would be continually rife, and the most conscientious college faculty would not be able to escape censure.

Dr. Buckingham defended the colleges, and doubted whether any State Board of Examiners could improve upon the present condition of affairs. The trouble is that only a small proportion of those who go to the colleges can be made professional men. They have neither an English education, nor professional instincts. No college training can make anything but quacks of such students.

Dr. Morgan fiercely attacked the idea of a Board of State Examiners. He also strongly condemned the plan of establishing chairs of dentistry in medical colleges, as tending directly toward dental quackery. The graduates would obtain just enough of dental knowledge to enable them to stick some tools in their saddle-bags, and go around the country pulling a tooth here, putting in an amalgam filling there, and, trying to ride both the dental and medical horses at once, they would of necessity come to the ground. Those who made a failure in medicine would fall back upon dentistry, and the smattering of dental knowledge which they obtained would help to make them medical failures, while they would not know enough of dentistry to make respectable or honest or reputable practitioners of that. Thus the plan would work evil to both professions, and to the colleges which adopted it.

Dr. Brophy said that if dentristry had any kinship with medicine, the proper place to teach it was in a medical college. As it was, medical graduates knew less of the teeth than of any tissue in the body, because they were taught nothing. They knew of but one remedy for tooth troubles, and that was promiscuous and wholesale extraction. He would have them taught something concerning the function and importance of the teeth, that they might not be extracting them through ignorance. If medical men were aware of what could be done in diseased conditions, they would counsel a proper care of the teeth. Again, medical men were in such ignorance that many diseases which might be directly traceable to tooth or oral lesions they treated as constitutional. Common exposure of the pulp of a tooth they regarded as neuralgia. Alveolar abscess they frequently treated as necrosis, and *vice versa.* Every dental practitioner knew of cases in which an encysted tooth, or the exposed end of a deciduous tooth had been treated as an osseous tumor. Cases of mistaken diagnoses, due to the ignorance of the physician, were of every-day occurrence. He would have these men taught enough of dental diseases to be able to properly diagnose them, and then refer them to the dentist, as the man best qualified to deal with them. That was what the Chicago idea meant—the more perfectly equipping of the physician, that he might the more intelligently co-operate with the dentist.

Dr. Allport desired to defend the Chicago college plan. The idea of trying to found a profession upon the segment of another profession is the supremest kind of nonsense. If dentistry is any part of medicine, if it is, as our code of ethics claims, a specialty in medicine, then it is utterly impossible that one should have a complete and comprehensive knowledge of it without a thorough grounding in the principles of the mother profession. We shall never be what we wish to be until we lay the foundations broad and deep. A separate profession cannot be reared with such a narrow substructure as a single stone out of another profession. The proper basis upon which to found dental practice is contained in the great principles of medical science.

Dr. Hunter said that the great mistake is in accepting students who are not properly educated and qualified for professional practice.

Dr. Noell offered a resolution providing for an association of Dental Colleges, with a view to fixing the qualifications of graduates. Upon being put to vote it was declared lost.

Dr. Taft read a paper upon Dental Literature. The United States, he said, had a larger number of dental journals than all the world besides. Our ten journals contain each month four hundred and twenty-five pages, or an aggregate of five thousand one hundred pages per year. The British journals contained one thousand six hundred and ninety-two pages per year; the German, one thousand nine hundred and eighty-four; the Italian and Spanish, one hundred and sixty-four; the French, nine hundred and forty-eight; making in all, four thousand eight hundred and twenty-four pages. Thus the dental periodical literature of the United States exceeded that of all the rest of the world by one hundred and seventy-six pages.

The paper was discussed in connection with the subject of dental literature generally.

The officers elect were installed, and President Goddard delivered an address on taking the chair. He closed by saying that he assumed the gavel feeling the consciousness of success.

A vote of thanks was moved for all those who had contributed to the prosperity of the meeting and the welfare of the members.

The President named as the Committee of Arrangements for the meeting next year, at Niagara Falls, Drs. W. C. Barrett, of Buffalo; C. R. Butler, of Cleveland; and George L. Field, of Detroit.

The Association then adjourned.

---

